# Supplementary material for: A novel mechanism for the loss of mRNA activity in lipid nanoparticle delivery systems
Source: Nat Commun. 2021 Nov 22;12:6777. doi: 10.1038/s41467-021-26926-0 (PMC8608879; doi:10.1038/s41467-021-26926-0)
Supplement: Supplementary file 2 — Reporting Summary [file 41467_2021_26926_MOESM2_ESM.pdf]

nature research

Corresponding author(s): Phillip White

Last updated by author(s): Sep 20, 2021

Reporting Summary

Nature Research wishes to improve the reproducibility of the work that we publish. This form provides structure for consistency and transparency in reporting. For further information on Nature Research policies, see our Editorial Policies and the Editorial Policy Checklist.

Statistics

For all statistical analyses, confirm that the following items are present in the figure legend, table legend, main text, or Methods section.

n/a	Confirmed	
☐	☒	The exact sample size (*n*) for each experimental group/condition, given as a discrete number and unit of measurement
☐	☒	A statement on whether measurements were taken from distinct samples or whether the same sample was measured repeatedly
☒	☐	The statistical test(s) used AND whether they are one- or two-sided<br>*Only common tests should be described solely by name; describe more complex techniques in the Methods section.*
☒	☐	A description of all covariates tested
☒	☐	A description of any assumptions or corrections, such as tests of normality and adjustment for multiple comparisons
☐	☒	A full description of the statistical parameters including central tendency (e.g. means) or other basic estimates (e.g. regression coefficient) AND variation (e.g. standard deviation) or associated estimates of uncertainty (e.g. confidence intervals)
☒	☐	For null hypothesis testing, the test statistic (e.g. *F*, *t*, *r*) with confidence intervals, effect sizes, degrees of freedom and *P* value noted<br>*Give P values as exact values whenever suitable.*
☒	☐	For Bayesian analysis, information on the choice of priors and Markov chain Monte Carlo settings
☒	☐	For hierarchical and complex designs, identification of the appropriate level for tests and full reporting of outcomes
☒	☐	Estimates of effect sizes (e.g. Cohen's *d*, Pearson's *r*), indicating how they were calculated

Our web collection on statistics for biologists contains articles on many of the points above.

Software and code

Policy information about availability of computer code

Data collection	BD FACSDiva version 8.0
Data analysis	PROSize 2.0 and FlowJo Software 10.4

For manuscripts utilizing custom algorithms or software that are central to the research but not yet described in published literature, software must be made available to editors and reviewers. We strongly encourage code deposition in a community repository (e.g. GitHub). See the Nature Research guidelines for submitting code & software for further information.

Data

Policy information about availability of data

All manuscripts must include a data availability statement. This statement should provide the following information, where applicable:

- Accession codes, unique identifiers, or web links for publicly available datasets
- A list of figures that have associated raw data
- A description of any restrictions on data availability

The authors declare that the data supporting the findings of this study are available within this Article and its Supplementary Information. Source data are provided with this paper.

Field-specific reporting

Life sciences study design

All studies must disclose on these points even when the disclosure is negative.

Sample size	Duplicate wells were assayed for in vitro expression based on experience with the reproducibility of the assay
Data exclusions	No data were excluded
Replication	All in vitro testing was completed in duplicate or triplicate with 1 replicate, unless otherwise stated
Randomization	Robustness of the HPLC, MS, and cell-based assays have been demonstrated, thus randomization was not required
Blinding	Techniques used to identify adduct (HPLC, differential analysis, in vitro expression) are unbiased and did not require blinding

Reporting for specific materials, systems and methods

We require information from authors about some types of materials, experimental systems and methods used in many studies. Here, indicate whether each material, system or method listed is relevant to your study. If you are not sure if a list item applies to your research, read the appropriate section before selecting a response.

Materials & experimental systems

n/a	Involved in the study	
☐	☒	Antibodies
☐	☒	Eukaryotic cell lines
☒	☐	Palaeontology and archaeology
☒	☐	Animals and other organisms
☒	☐	Human research participants
☒	☐	Clinical data
☒	☐	Dual use research of concern

Methods

n/a	Involved in the study	
☒	☐	ChIP-seq
☐	☒	Flow cytometry
☒	☐	MRI-based neuroimaging

Antibodies

Antibodies used	Proprietary custom target/antibody pair
Validation	Primary antibody validation is not relevant to the study, all experiments shown in this study are examining relative expression

Eukaryotic cell lines

Policy information about cell lines

Cell line source(s)	ATCC for BJ fibroblasts and HeLa cell lines
Authentication	Cell lines were authenticated by Short Tandem Repeat (STR) profiling
Mycoplasma contamination	All cells tested negative
Commonly misidentified lines (See ICLAC register)	No commonly misidentified cell lines were included

Flow Cytometry

Plots

Confirm that:

☒ The axis labels state the marker and fluorochrome used (e.g. CD4-FITC).

☒ The axis scales are clearly visible. Include numbers along axes only for bottom left plot of group (a 'group' is an analysis of identical markers).

☒ All plots are contour plots with outliers or pseudocolor plots.

☒ A numerical value for number of cells or percentage (with statistics) is provided.

Methodology

Sample preparation	Transfected HeLa cells were , fixed, permeabilized and stained with proprietary primary antibody
Instrument	BD LSRFortessa Flow Cytometer
Software	Data collection utilized BD FACSDiva (version 8.0) and analysis was performed using FlowJo (version 10.4)
Cell population abundance	For this analysis, the assay was run to a target 10,000 counts per sample
Gating strategy	Gating was performed by targeting 5% of the fluorescence of the non-translating mRNA negative control

☒ Tick this box to confirm that a figure exemplifying the gating strategy is provided in the Supplementary Information.
